# Primary health services at district level in South Africa: a critique of the primary health care approach

**DOI:** 10.1186/1471-2296-13-67

**Published:** 2012-07-02

**Authors:** Sunitha Dookie, Shenuka Singh

**Affiliations:** 1 School of Health Sciences, University of KwaZulu-Natal, Private Bag X54001, Durban, 4000, South Africa

**Keywords:** Primary health care, District health system

## Abstract

**Background:**

The rhetoric of primary health care philosophy in the district health system is widely cited as a fundamental component of the health transformation process in post-apartheid South Africa. Despite South Africa’s progress and attempts at implementing primary health care, various factors still limit its success.

**Discussion:**

Inconsistencies and poor understanding of primary care and primary health care raises unrealistic expectations in service delivery and health outcomes, and blame is apportioned when expectations are not met. It is important for all health practitioners to consider the contextual influences on health and ill-health and to recognise the role of the underlying determinants of ill-health, namely, social, economic and environmental influences. The primary health care approach provides a strong framework for this delivery but it is not widely applied.

There is a need for renewed political and policy commitments toward quality primary health care delivery, re-orientation of health care workers, integration of primary health care activities into other community-based development, improved management skills and effective coordination at all levels of the health system. There should also be optimal capacity building, and skills development in problem-solving, communication, networking and community participation.

**Summary:**

A well-functioning district health system is required for the re-engineering of primary health care. This strategy requires a strong leadership, a strengthening of the current district heath system and a greater emphasis on health promotion, prevention, and community participation and empowerment.

## Background

The rhetoric of primary health care philosophy in the district health system is widely cited as a fundamental component of the health transformation process in post-apartheid South Africa [1,2]. The principles of primary health care (PHC) such as efficiency and effectiveness in health service delivery; and equitable distribution of health services are identified as core elements with the potential to contribute to improved community health through properly coordinated district health systems [3].

The major challenges facing primary health care include ‘adequate political, financial, human and material commitments; optimal use of available resources; changing management techniques including decentralization; and ensuring effective community participation and intersectoral collaboration’ [4]). The historical imbalances in health care delivery in South Africa, coupled with the changing patterns of disease and the complex burden of communicable and non-communicable disease places a huge strain on the public health service.

This paper outlines the challenges facing district primary health care delivery in South Africa, defines primary care and primary health care, and identifies mechanisms that could assist health planning efforts at the micro level (individual/community), and the macro and meso (national/provincial/district health system) levels of health service delivery.

## Discussion

### PHC at district level

The district health system has been sanctioned as the vehicle for the implementation of primary health care at the community level [5]. However the process of implementing and integrating the health system at district level has been slow and inconsistent with some areas reflecting well functioning health units while other areas have fragmented and poorly coordinated primary health care delivery systems [6]. Inequalities in the coverage and quality of health services, inherent inequities in resource allocation, coupled with the historical burden of disease indicates that provinces and districts are not at the same level of health care delivery. This is further complicated with the burden of the HIV/AIDS pandemic, emerging infectious and non communicable diseases that places a severe strain on scarce resources. This imbalance in health care delivery, together with a curative driven health system; lack of commitment to preventive and promotive services; and ineffective leadership, will ensure that health inequalities and unmet health need will persist [7-9].

Although the South African experience in implementing primary health care does show some positive impact, in terms of increased access in comparison to the pre-Apartheid health service delivery (pre 1994), various factors still limit its success [10,11]. Some of the identified gaps in implementation include resource constraints; migration of professionals; the unequal distribution of personnel in public and private sectors; low skills levels; poor staff motivation and the lack of managerial capacity [10,12-14]. The critical shortage of key health personnel is an important barrier in the provision, implementation and sustainability of district health services. The widening gap between the public and private health sectors, specifically with regard to coordination and resource allocation are also areas of concern [7].

### Defining primary care and primary health care

Some of the factors that complicate primary health care service delivery would be the definition and scope of primary care and primary health care. The concepts of primary care and primary health care have been widely used interchangeably however there are inherent differences between these concepts [15,16]. The multiplicity of interpretations and efforts to distinguish primary care from primary health care justifies why there is no single model for primary health care, and thus, the problems experienced with implementation [17].

### Primary care

Primary care refers to services provided by general practitioners, nurses or other allied health professionals and is regarded as the first point of entry to the health system. Primary care is oriented towards disease prevention and focuses on individuals and families. This level of care allows for early diagnosis and treatment management, and referral to secondary and tertiary care, thereby providing the potential for continuity of care. Primary disease prevention focuses on health risks, and health education and/or other preventive strategies are directed to the individual or family, rather than the social or environmental factors that influence disease progression. Although health educational strategies are directed to individuals or families, there can also be community engagement, eg, consultations with consumer groups health needs, perceptions on health service delivery, health education with school children, etc [18].

#### Primary health care

Primary health care, on the other hand, is a public health strategy derived from the social model of health and is based on the philosophy that health gains are better obtained when people’s basic needs are met first [18]. The fundamental principles of primary health care at the operational level are unique to the specific circumstances or system. Thus the underlying social determinants on ill-health such as access to basic living conditions, unemployment, etc are important factors to consider in the primary health care strategy. The strength of primary health care is to respond to the local needs of individuals, families and populations through a comprehensive, inter-sectoral approach that focuses on communities as the unit of intervention. In essence, primary health care provides a connection between health and health care, by linking this to social and economic systems. The principles of the primary health care approach include equity in health service delivery, access to affordable and appropriate services, empowerment of people, and sustainability of service provision. [18,19].

From a conceptual perspective, community-oriented primary care (COPC) is seen as the link between the concepts of Family Medicine and Primary Health Care. This provides a ‘planned integration of primary care with public health in a defined community’, where priority health planning ensures that the focus is on health and not just on illness. Reid (2010) postulates a population-based approach, within a defined community, using the entire primary care team to address issues of access and quality of care. This however requires a re-definition of roles and effective leadership [20].

### The way forward

#### Disease prevention and health promotion

These factors outlined have important implications for the delivery of district health services. Inconsistencies and poor understanding of primary care and primary health care raises unrealistic expectations in service delivery and health outcomes, and blame is apportioned when expectations are not met. At the micro level of health delivery, it is important for all health practitioners to consider the contextual influences on health and ill-health and to recognise the role of the underlying determinants of ill-health, namely, social, economic and environmental influences. Comprehensive primary care using strategies of the primary health care approach could focus on integrated health care delivery, where individuals and communities are managed holistically. This includes the recognition that chronic diseases as a result of lifestyle practices, will result in a number of disease presentations eg. unhealthy dietary practices are linked to obesity, dental caries, cardiac complications, diabetes, etc. Thus strategies to address the risks to unhealthy lifestyle practices must recognise the role of optimal oral health and nutritional status in improving overall health outcomes [21]. These would include the need to create supportive environments to promote the selection of healthier choices. The primary health care approach provides a strong framework for this delivery but it is not widely applied. The primary health care framework also allows for assessment of the quality, appropriateness and impact of service delivery, the identification of gaps, and research development. Healthy living and family self care initiatives will make significant contributions to foster individual and community empowerment if there is coordinated effort to focus on disease prevention and health promotion.

The World Health Assembly resolution on primary health care (2009) reiterates ‘the importance to reorganize disease- or health problem-specific (vertical) actions through comprehensive (horizontal) primary health care [19,22]. The focus of this resolution is ‘to train and retain adequate numbers of health workers, with appropriate skill mix, including primary health care nurses, midwives, allied health professionals and family physicians, able to work in a multidisciplinary context, in cooperation with non-professional community health workers in order to respond effectively to people’s health needs [19,22].

#### Strategic organisational planning

A dynamic health system that is able to monitor and respond to unforeseen challenges, including demographic transitions and changing health needs, is required. At the macro and meso level of health care delivery, it is important that health system development be coordinated with areas of development, such as social, economic and environmental development. Thus re-engineering of primary health care and the implementation of the National Health Insurance System could provide a mechanism to redress current health inequalities in South Africa. [13]. However Sanders et al (2011) argue that the impact of neoliberal economic policies and the broader impact of economic forces on primary health care delivery must be considered. Regulation of the market is required if revitalisation of primary health care and progress towards health equity is to be realized [23].

Primary health care requires structural re-organisation, multidisciplinary team approach with clear lines of accountability, clear referral patterns in a two-way direction, improved access to health insurance to improve health coverage, and developing effective public-private partnerships. The universal core packages of health care should be evidence-based, cost-effective and appropriate to local needs [4,22].

Policy development on integrated health care generally fails to guide the translation of policy rhetoric into health action. The delays in health integration are largely due to the current lack of clear direction and accountability at district and institutional levels [24]. Effective leadership is required in terms of policy formulation and translation into practice, development of monitoring tools for assessment of the health system including the burden of disease, utilization of health services, and effectiveness of health interventions. There is a need for renewed political and policy commitments toward quality primary health care delivery, re-orientation of health care workers, integration of primary health care activities into other community-based development, improved management skills and effective coordination at all levels of the health system. There should also be optimal capacity building, and skills development in problem-solving, communication, networking and partnership formation. Apart from capacity building, there should be incentives to motivate health personnel and support career development [4,22].

#### Intersectoral collaboration and community participation

Other levels of care, such as ambulatory care (home-based, community-based) should be explored for appropriateness and effectiveness in providing continuity in care. There should be strengthening of the decentralization process in district health services, fostering of community participation and engagement in quality assurance. The role of non-governmental organizations, traditional medicine and other stakeholders in community development must be considered.

There is a significant reliance on traditional medicine in South Africa. Therefore the role of pluralistic health care (complementary use of allopathic services should be explored to identify mechanisms to support the health system and increase health coverage in a manner that is socially and culturally acceptable and appropriate to communities [7]. Strategic resource allocation on an equitable basis would be required to maximize health benefits. There should be a focus on cost-effectiveness and efficiency indicators. Policies on the selection, procurement, distribution and quality of appropriate technology, including the availability of drugs, should be explicitly translated into effective practice [4,7,22].

Community participation is central to empower individuals and communities to be part of their health decision making processes. Community participation and empowerment can be best fostered through transparent policy decisions and equitable resource allocation that recognizes community needs and priorities.

## Summary

The philosophy of primary health care is an important component for the health transformation process in South Africa, however, a well-functioning district health system is required for the re-engineering of primary health care. This approach requires strong leadership, strengthening of the current district heath system with a greater emphasis on health promotion and prevention, a recognition of the role of traditional/complementary health care, together with commitments in intersectoral collaboration, and community participation and empowerment.

## Competing interests

The authors declare that they have no competing interests.

## Authors’ contribution

SD and SS conceptualized the original paper. SD was responsible for writing the paper. SS was responsible for re-conceptualising and writing the amended paper. Both authors read and approved the manuscript.

## Pre-publication history

The pre-publication history for this paper can be accessed here:

http://www.biomedcentral.com/1471-2296/13/67/prepub
